# Comparative Proteomic Analysis of the Stolon Cold Stress Response between the C_4_ Perennial Grass Species *Zoysia* *japonica* and *Zoysia* *metrella*

**DOI:** 10.1371/journal.pone.0075705

**Published:** 2013-09-26

**Authors:** Jiping Xuan, Yufeng Song, Hongxiao Zhang, Jianxiu Liu, Zhongren Guo, Yuelou Hua

**Affiliations:** 1 Institute of Botany, Jiangsu Province and the Chinese Academy of Sciences, No.1 Qianhu Houcun, Zhongshanmenwai, Nanjing, Jiangsu Province, PR China; 2 College of Life and Science, Nanjing Agricultural University, No.1 Weigang, Nanjing, PR China; 3 College of Agriculture, Henan University of Science and Technology, No.263 Kaiyuandadao, Luoyang, Henan Province, PR China; Lawrence Berkeley National Laboratory, United States of America

## Abstract

Zoysiagrass, the most cold-tolerant grass among the warm-season turfgrasses, is often used as a model species for isolating cellular components related to cold stress. To understand the proteomic responses to cold stress in zoysiagrass stolons, we extracted stolon proteins from *Zoysia* *japonica* , cv. Meyer (cold-tolerant) and *Z* *. metrella* , cv. Diamond (cold-sensitive), which were grown with or without cold treatment. Approximately 700 proteins were resolved on 2-DE gels, and 70 protein spots were differentially accumulated. We further observed that 45 of the identified proteins participate in 10 metabolic pathways and cellular processes. A significantly greater number of proteins accumulated in the Meyer than in the Diamond and 15 increased proteins were detected only in the Meyer cultivar under cold stress. Furthermore, we propose a cold stress-responsive protein network composed of several different functional components that exhibits a balance between reactive oxygen species (ROS) production and scavenging, accelerated protein biosynthesis and proteolysis, reduced protein folding, enhanced photosynthesis, abundant energy supply and enhanced biosynthesis of carbohydrates and nucleotides. Generally, the cold-tolerant Meyer cultivar showed a greater ROS scavenging ability, more abundant energy supply and increased photosynthesis and protein synthesis than did the cold-sensitive Diamond cultivar, which may partly explain why Meyer is more cold tolerant.

## Introduction

Low temperature is one of the most serious types of environmental stress and can reduce growth and cause rolling and withering of plant leaves. It is particularly important for plant biologists to understand the molecular mechanisms underlying the plant response to low temperature [1]. Plants usually present several strategies, including gene regulation, to defend against cold stress. Many studies have focused on gene expression profiles during cold stress, because cold-responsive proteins are likely to be involved in cold tolerance [2]. Several approaches, including the identification of novel responsive genes, determination of their expression patterns, and understanding their functions in stress responses, have been applied to develop effective engineering strategies that may lead to greater stress tolerance [3]. Recently, with the advent of proteomics, gene expression during cold stress and acclimation has been studied via both transcriptomic and proteomic strategies. Microarray analyses have also shown that cold alters the expression of myriad genes [3,4], whereas proteomic approaches have identified no more than 150 proteins related to cold tolerance in *Arabidopsis* and rice [5,6,7,8].

Proteomics, which combines two-dimensional electrophoresis (2-DE) with mass spectrometry (MS) and improving databases, is recognized as a powerful approach for comparing proteomes under various stress conditions. Recently, a proteomics approach has been applied to study plant stress responses. Several studies examining plant transcriptomes during cold stress have been performed Cui et al. [1] and Yan et al. [8] investigated the proteins that are involved in the cold stress response and recovery from cold stress in rice leaves; Lee et al. [9] anayzed the *Arabidopsis* cold-responsive proteins, Rabbani et al. [10] monitoring the rices protein expression under cold, Hashimoto and Komatsu [11] investigated the protein expression of rice seedlings leaf blades, leaf sheaths and roots under cold stress and Wang et al. [12] described the proteins that are associated with cold stress in moss gametophores. Kosmala et al. [13] reported significant differences in the protein accumulation profiles between high frost and low frost *Festuca* *pratensis* plants during cold acclimation, and one-half of the differentially accumulated proteins constituted components of the photosynthetic apparatus. These results have provided useful information for understanding cold stress-responsive proteins.

Zoysiagrass (*Zoysia* spp. Willd.) is a widely used, environmentally friendly warm-season turfgrass species that is indigenous to the nations of the western Pacific Rim. Cold stress is the primary limiting factor for the distribution of zoysiagrass in transitional and temperate regions. However, zoysiagrass exhibits greater freezing tolerance than other warm-season turfgrasses [14], and the injuries incurred during the winter vary widely among zoysiagrass genotypes [15,16,17]. The physiological basis for these differences has only partially been explored [18,19]. The physiological changes that occur during cold acclimation in plants include increases in the concentrations of sugars, organic acids, proline, soluble proteins and polar lipids [20,21,22,23,24]. In a previous study by our group, the zoysiagrass cultivars Meyer ( *Zoysia* *japonica* ) and Diamond ( *Z* *. metrella* ) were identified as being freeze-tolerant and freeze-sensitive, respectively [17]. To fully understand the regulatory mechanism involved in the cold tolerance of zoysiagrass, studies performed at the protein level using a proteomic approach are required. In the present study, the differences in the proteins that accumulate in the stolon of these two zoysiagrass cultivars and show differential freeze tolerance were identified based on 2-DE and peptide mass fingerprints (PMFs) obtained via matrix-assisted laser desorption/ionization-time-of-flight (MALDI-TOF) MS to characterize cold-stress responsive proteins and better understand the mechanisms involved in the cold stress response. Based on the obtained proteomic data, the molecular mechanisms involved in cold stress responses in zoysiagrass stolons are discussed. Our results should be helpful for better understanding the acclimation mechanism of *Zoysia* spp. under cold stress.

## Materials and Methods

### Plant material and cold treatments

The stolons of *Zoysia* *japonica* cv. Meyer and *Zoysia* *metrella* cv. Diamond, which were screened and identified as a freeze-tolerant and a freeze-sensitive cultivars, respectively, were collected from field plots from the turfgrass research farm at Institute of Botany, Jiangsu Province and the Chinese Academy of Sciences (Xuanwu, Nanjing, China), and vegetatively propagated in plastic pots (28 cm deep and 23 cm in diameter) filled with 90% sand and 10% compound fertilizer (15:15:15 N:P:K). During the plant establishment period, the plants were watered as needed and fertilized twice a week with full-strength Hoagland’s nutrient solution [25]. The plants were maintained in a greenhouse for 28 d with natural sunlight with photosynthetically active radiation (PAR) in the range of 500-1,000 µmol m^-2^s^-1^ and an average day/night temperature of 32/28 °C. Then, the plants were moved to a growth chamber set at 28/22 °C (day/night temperature) with 75% relative humidity, 300 µmol m^-2^s^-1^ PAR and a 14-h photoperiod. The plants were allowed to acclimate to the growth chamber conditions for 14 d before treatments were imposed.

After 14 d of acclimation, half of the plants were transferred to a growth chamber set at 8/2 °C (day/night temperature, cold stress treatment), while the other plants remained in the growth chamber set at 28/22 °C (day/night temperature, control) for 28 d. Each treatment was replicated in three pots for each cultivar. During the treatment period, the plants were fertilized once a week with full-strength Hoagland’s nutrient solution [25].

### Electrolyte leakage measurement

**Figure 1:**
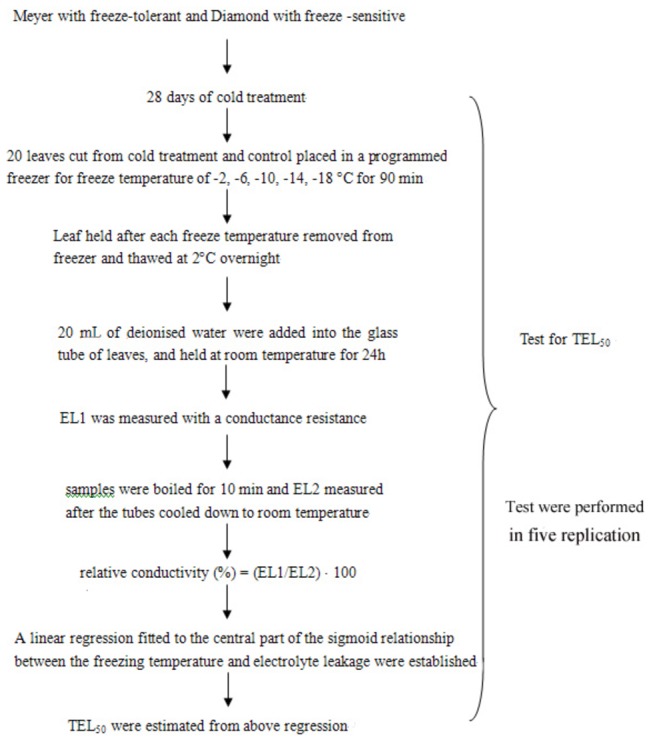
A schematic diagram of chronological order of the cold-tolerance measurement of two zoysiagrass under 28-d cold stress compared to those under non cold stress (control)

A schematic diagram of the experimental process to examine cold-tolerance of zoysiagrass under 28-d cold stress compared to those under non cold stress (control) is presented in Figure 1. Estimation of the TEL_50_ (temperature causing 50% electrolyte leakage) was performed on the two cultivars on both cold control and cold stress treatment (after 28-d of cold stress) to determine their level of freeze tolerance. Leaves were cut from the plants (20 leaves for each freezing temperature of -2, -6, -10, -14 and -18 °C) and placed in a programmed freezer (Polyscience 9610, Polysciences, Inc., U.S. Corporate Headquarters, Pennsylvania, USA) with a temperature error of ± 0.1 °C. The freezing temperature was decreased at a rate of 4 °C h^-1^ and then held at each freezing temperature for 90 min. The samples were then removed from the freezer and thawed at 2 °C overnight. The cell membrane damage in the leaves collected after freezing was determined by measuring the electrical conductivity of tissue extracts, as described in detail by Xuan et al. [17]. The TEL_50_ values were estimated from a linear regression fitted to the central (linear) part of the sigmoid relationship between the freezing temperature and electrolyte leakage at the five temperatures.

### Tissue preparation and extraction of zoysiagrass stolon proteins

After 28 d of cold stress, whole stolons were harvested from zoysiagrass plants from both the control and cold stress treatments [23]. All harvested stolons were frozen and stored in liquid nitrogen after washing off the soil and removing the leaves, roots and stems. The samples were then stored in liquid nitrogen.

For each protein sample, 1 g of plant material was used. The protein extraction procedure was based on a TCA/acetone method [26]. The stolons were ground with a mortar and pestle in liquid nitrogen to obtain a fine powder and suspended with 20 mL of ice-cold extraction buffer (10% w/v TCA in acetone containing 0.07% w/v DTT), incubated at -20 °C for 2 h and centrifuged for 15 min at 16,000 ×*g*. The pellets were subsequently resuspended in 20 mL of acetone containing 0.07% w/v DTT, incubated at -20 °C for 1 h and centrifuged for 15 min at 12,000 ×*g*. This step was repeated three times, and the pellets were then lyophilized. The resulting powder was solubilized in lysis buffer (9 M urea, 35 mM Tris, 4% w/v CHAPS, 0.2% w/v Bio-lytes pH 4–7 IPG buffer, 65 mM DTT, 1 mM PMSF), followed by centrifugation for 15 min at 12,000 × *g*. The proteins in the supernatant were precipitated by adding four volumes of ice-cold acetone, followed by incubation at -20 °C for at least 2 h and centrifugation for 15 min at 12,000 ×*g*. The pellets were then dissolved in rehydration buffer (8 M urea, 65 mM DTT, 4% w/v CHAPS, 0.2% w/v pH 4–7 IPG buffer). The protein concentrations were determined using the modified Bradford assay and ovalbumin as a standard [27].

### 2-DE, gel staining and image analysis

For each replicate, aliquots of proteins (200 µg) were mixed with rehydration solution (8 M urea, 4% CHAPS, 0.2% IPG buffer, pH range 4-7, 0.001% bromophenol blue and 65 mM DTT) in a final volume of 320 µl and used for 2-DE. For first-dimension isoelectrofocusing (IEF), 17 cm Bio-Rad gels with linear pH range of 4-7 were used. Rehydration and focusing were performed in a PROTEAN IEF Cell (Bio-Rad) at 50 µA per strip at 20 °C, applying the following program: 12 h of rehydration at 50 V, 1 h at 250 V, 1 h at 1,000 V and 5 h at 10,000 V with focusing for a total of 60,000 V·h. After IEF, the gel strips were equilibrated for 15 min in 5 mL of SDS equilibration buffer solution (0.375 M Tris-HCl pH 8.8, 6 M urea, 20% v/v glycerol, 2% w/v SDS and 2% w/v DTT), followed for 15 min with the same buffer but containing 2.5% w/v iodoacetamide instead of DTT.

After equilibration, the proteins were separated in the second dimension (SDS-PAGE) using 12.5% SDS-polyacrylamide gels (1.5mm×250 mm×200 mm) sealed with 0.5% agarose. Electrophoresis was performed at 75 V for 1 h, followed by 150 V for 9 h using a PROTEAN Plus Dodeca cell apparatus (Bio-Rad). The obtained protein spots were visualized via MS-compatible silver staining [28]. 

The signal was visualized via silver staining. The stained gels were scanned at a 300 dots per inch (dpi) resolution with a UMAX Powerlook Ш scanner (UMAX Technologies, USA), and image and data analyses of the gels were performed using PDQuest software (Version 8.0; Bio-Rad). Spot quantity normalization was conducted in the ‘total quantity of valid spots’ mode for possible staining differences between gels. Duplicate 2-DE gels were run for each treatment from three independent tissue extractions, considering the two cultivars from two species (Meyer and Diamond) and two treatments (control and cold treated), 12 silver-staining gels were scanned and analyzed. And only those spots with significant and reproducible changes were considered to represent differentially expressed proteins. The results for the control and cold stress samples were analyzed for differences using the analysis of variance (ANOVA) and Student’s t-test with a significance level of 95%. Protein spots were selected when a significant expression variation of 2.0-fold or greater existed as compared to the control in at least one cultivar. Then, Boolean analysis sets were created between the statistic sets and the quantitative or qualitative sets. Selected protein spots were manually excised from the gels for MALDI-TOF MS analysis.

### In-gel digestion of protein and MALDI-TOF-MS analysis

The differentially accumulated proteins were excised manually and placed in 0.5mL Eppendorf tubes for in gel digestion.

The silver-stained proteins were destained using chemical reducers to remove the silver, as described previously, with the following critical modifications [29]. The gels were washed twice with Milli-Q water (Millipore, Bedford, MA), destained with fresh solution (a 1:1 mixture of 15 mM potassium ferricyanide and 50 mM sodium thiosulfate) and occasionally vortexed. The brownish color disappeared after 1-2 minutes, and the gel bands were then rinsed a few times with water to stop the reaction. Next, 200 mM ammonium bicarbonate was added to cover the gel for 5 min, followed by repeated dehydration with changes of acetonitrile until the gel pieces turned opaque white; the gel was then dried in a vacuum centrifuge.

Enzymatic digestion was performed with 20 µg/mL trypsin (with 40 mmol ammonium bicarbonate and 9% (v/v) acetonitrile) with incubation for 45 min in an ice bath, and the supernatant was subsequently removed. Then, 40 mM of an ammonium bicarbonate solution containing 9% (v/v) acetonitrile was added to cover the gel, followed by incubation for 20 h at 37 °C. Following enzymatic digestion, the resultant peptides were extracted by two 15-min incubation in an extraction solution containing 0.1% trifluoroacetic acid and 60% acetonitrile. The extracts were dried in a vacuum centrifuge and the dried peptides were re-dissolved in 0.1% trifluoroacetic acid to remove impurities and then concentrated with ZipTip (Millipore Corp., Bedford, USA).

The peptide solution in 35% acetonitrile/0.1% trifluoroacetic acid was saturated with α-cyano-4-hydroxycinnamic acid and then air dried on an MS sample plate. A MALDI-TOF MS analysis was conducted with a REFLEX III MALDI-TOF/TOF mass spectrometer (Bruker-Daltonics, Leipzig, Germany) in the positive-ion reflector mode. Calibration was performed using a standard peptide mixture. The MS data were collected from monoisotopic peaks falling in the m/z range of 750–4000 Da with a signal: noise ratio >10. The peaks resulting from trypsin autolysis and keratin contamination were excluded from the mass list using Peakerazor (GPMAW, General Protein/Mass Analysis for Windows, Lighthouse Data, Odense, Denmark; http://www.gpmaw.com).

### PMF analysis

Database searches were performed using the MASCOT program (http://www.matrixscience.com). The database was set to National Center Biotechnology Information (NCBI) non-redundant database (24070523 sequences/ 8281664780 residues for spots analyzed in April 2010), with a Viridiplantae filter (1272944 sequences for spots analyzed in April 2010) using the Mascot search engine (Mascot Daemon v. 2.2.2, Mascot Server V. 2.2.03, Matrix Science). The search type was PMF. The other parameters for searching were deﬁned as follows: trypsin enzyme, fixed modifications of cysteine as carbamidomethylated, variable modifications of methionine as oxidized, peptide charge state of H^+^, peptide tolerance of 100 ppm, and up to 1 missed enzymatic cleavage. Base on the MASCOT probability analysis (P≤0.05), only significant hits were accepted for identification of the protein samples.

## Results

### Analysis of zoysiagrass freeze tolerance under cold stress

**Figure 2:**
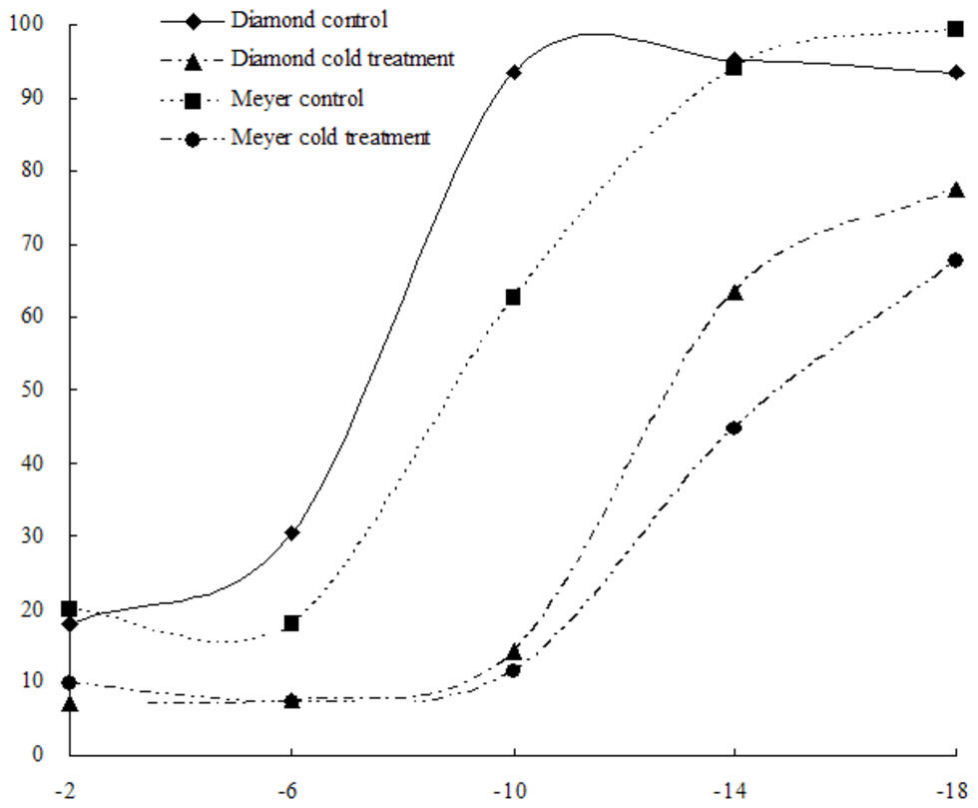
Sigmoidal curve between the freezing temperature and the relative electrolyte leakage data from a pilot study of the two zoysiagrass cultivars of both cold control and cold stress treatment (after 28-d cold stress treatment). Points represent the mean of five replications.

The plasma membrane not only separates the cytoplasm from the extracellular environment but also acts as the primary site for sensing and responding to environmental changes. Maintaining the integrity of the plasma membrane is necessary for an organism to survive. However, different degrees of stress often damage the membrane, resulting in leakage. Measuring electrolyte leakage is straightforward and is widely used to detect membrane damage. Here, we assayed the relative electrolyte leakage (REL) from the two zoysiagrass cultivars of both cold control and cold treatment (after 28 days at 8/2°C cold stress treatment) at particular freezing time points. As shown in Figure 2, in the control plants, REL increased slightly when the temperature dropped from -2 °C to -6 °C. When the temperature decreased further, from –6 °C to either -10 °C (Diamond) or -14 °C (Meyer), REL increased sharply, reaching a maximum at either -10°C (Diamond) or -14 °C (Meyer), followed by stable REL at the lowest cold temperature point. After 28 d under cold stress treatment, REL increased slightly when the temperature dropped from -2 °C to -10 °C. Then, when the temperature decreased below -10 °C, REL began to increase sharply and did not reach a maximum until -18 °C. As shown in Figure 2, the curve of the freezing temperature versus electrolyte leakage for Meyer was smoother than for Diamond, confirming that Meyer exhibited superior cold resistance compared to Diamond.

The freeze tolerance (TEL_50_) of the zoysiagrass cultivars decreased after cold acclimation from -5.9 °C to -11.9 °C (Diamond) and from -9.0 °C to -16.2 °C (Meyer). To study the later consequences of cold treatment on protein levels, 2-DE was used to display and compare stolon proteins from two different freezing-tolerant zoysiagrass cultivars from cold-treated and untreated plants.

### Effects of cold stress on protein accumulation

**Figure 3:**
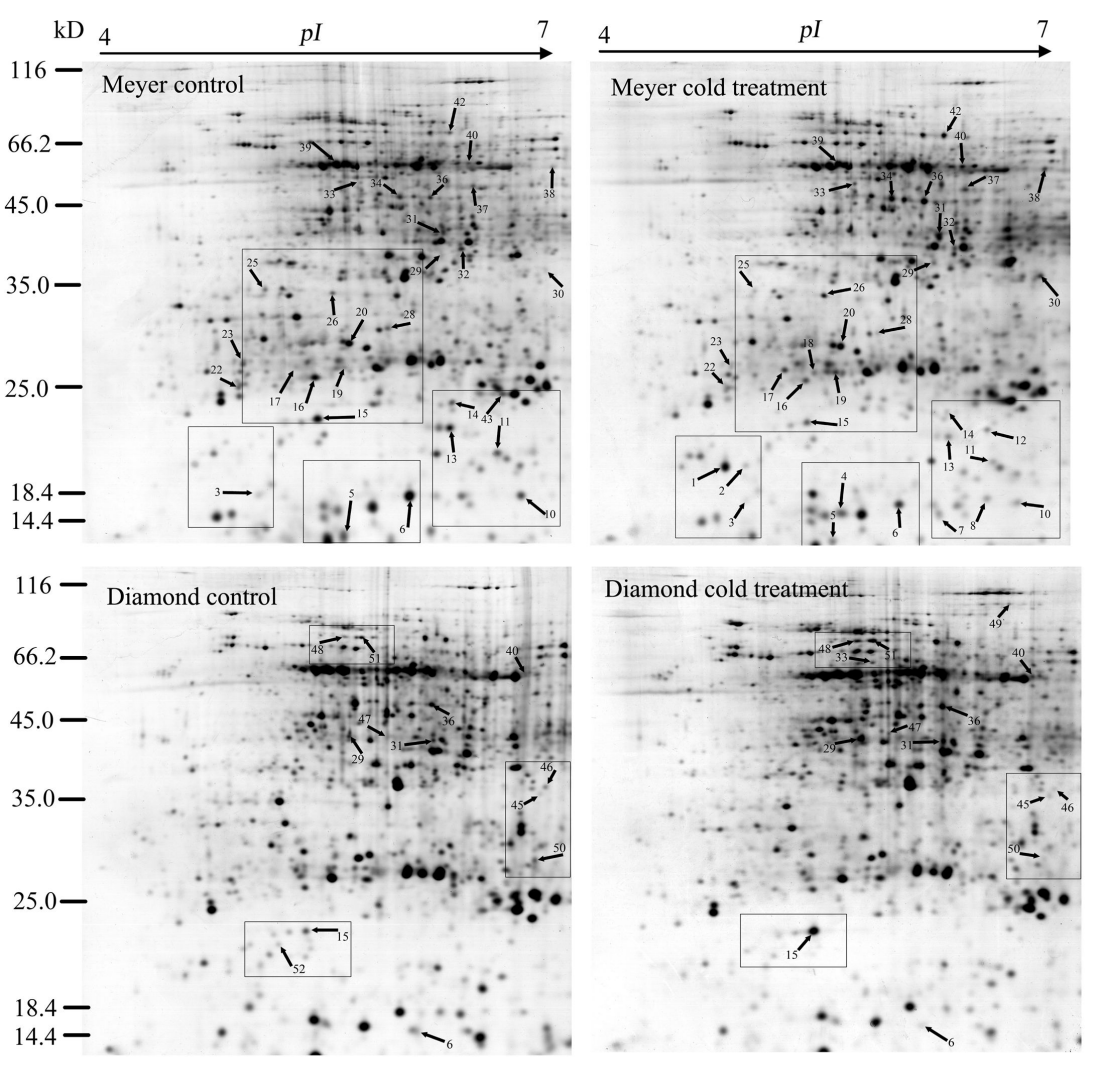
2-DE gels of stolon extracts from two zoysiagrass cultivars subjected to a control or cold stress treatment for 28 d. Silver-stained gels. Protein spots were assigned arbitrary identifiers. The framed regions are enlarged in Figure 4.

**Figure 4:**
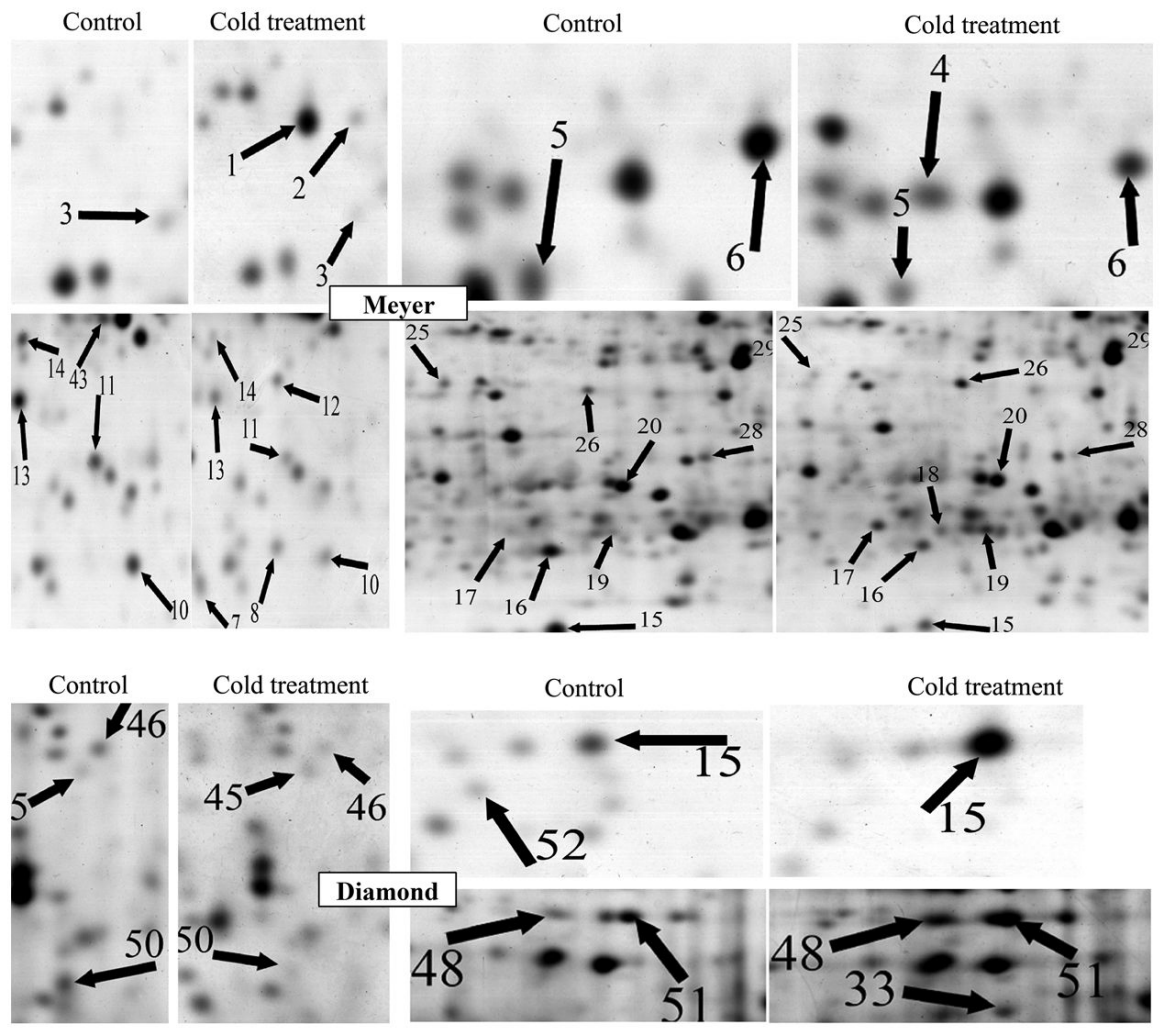
Comparison of some of the proteins that were differentially expressed in response to the cold treatment. The protein spot numbers are as in Figure 3 and Table 1. All enlargements are from silver-stained gels. Differentially expressed proteins are indicated with arrows.

In zoysiagrass, the stolon is an important organ that is involved in overwintering. Total proteins were extracted from the stolons of control and cold-treated zoysiagrass plants and separated on 2-DE gels. The protein maps produced from three independent protein extractions showed a high reproducibility based on analysis using PDQuest software. Figure 3 shows a representative gel image of proteins extracted from the control and cold-treated stolons. Approximately 700 protein spots were reproducibly detected using PDQuest 8.0 software within each analyzed gel (Figure 3). Seven typical regions are enlarged in Figure 4.

**Figure 5:**
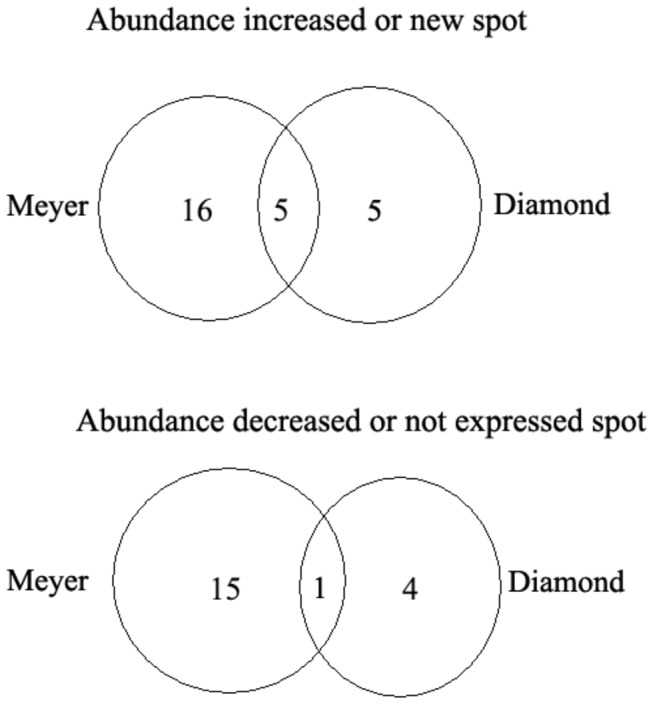
Venn diagram illustrating the expression patterns of cold stress-responsive proteins in zoysiagrass stolons. Among the increased or new spots, 16 spots (spot 1, PR; spots 2, 8 and 32, Rubisco large subunit; spot 4, NDPK; spot 7, Ndh; spot 12, proteasome α3 subunit; spot 17, APX; spot 18, cellulose synthase; spot 19, ATPase α subunit; spot 26, translation initiation factor; spot 30, phosphoglycerate kinase; spot 34, protein kinase; spot 37, NBS-LRR resistance protein RGH2; spot 38, ATP synthase β subunit and spot 42, CAT) were detected only in Meyer, 5 spots (spot 29, ADP-glucose pyrophosphorylase large subunit; spot 31, phosphoglycerate kinase; spot 33, enolase; spot 36, adenylate kinase family protein and spot 40, elongation factor Tu) were detected in both genotypes; and 5 spots (spot 15, expressed protein; spot 47, protein phosphatase 2c protein; spot 48, auxin response factor 1; spot 49, phosphoenolpyruvate carboxylase and spot 51, myosin heavy chain-related protein) were found only in Diamond; Among the decreased or non-expressed spots, 15 spots (spot 3, vacuolar ATP synthase G3; spot 5, prefoldin subunit 1; spot 10, Tr-h; spots 11 and 23, cytochrome P450 protein; spot 13, APX; spot 14, prolyl 4-hydroxylase, putative; spot 15, expressed protein; spot 16, Rubisco small subunit; spot 20, flavanone-3-hydroxylase; spot 22, PSF2; spot 25, dihydroflavonol-4-reductase; spot 43, bZIP transcription factor; spot 28, pyruvate dehydronase and spot 39, f-box protein) were detected only in Meyer, 1 spot (spot 6, RAB protein) was decreased in both genotypes; and 4 spots (spot 45, SNF1-related protein kinase; spot 46, 1-amino-cyclopropane-1-carboxylate synthase 8; spot 50, dihydropyrimidinase; and spot 52, Rubisco small subunit) were decreased only in Diamond. One spot (spot 15, Expressed protein) increased in abundance in Diamond but decreased in Meyer.

A quantitative image analysis revealed 70 protein spots that exhibited a fold change of more than 2.0 in their volume in at least one cultivar, and 45 protein spots were identified through MALDI-TOF MS and PMF (Table 1). Among these 45 protein spots, 26 spots (spots 1, 2, 4, 7, 8, 12, 15, 17-19, 26, 29-34, 36-38, 40, 42, 47-49 and 51) showed increased abundance or were newly accumulated, whereas 20 spots (spots 3, 5, 6, 10, 11, 13-16, 20, 22, 23, 25, 28, 39, 43, 45, 46, 50 and 52) presented decreased abundance or no accumulation in at least one genotype; the abundance of one spot (spot 15) decreased in Meyer, but increased in Diamond (Figure 5 and Table 1). In the group of spots showing a decline in abundance or no accumulation under cold stress, 1 spot (spot 6) decreased in both genotypes, while 14 spots (spots 3, 5, 10, 11, 13-16, 20, 22, 23, 25, 28, 39 and 43) only decreased or were not expressed in Meyer (Figure 5, Table 1). Among the 26 spots showing an increase in abundance or new accumulation, 5 spots (spots 29, 31, 33, 36 and 40) increased or were newly accumulated in both genotypes, 15 spots (spots 1, 2, 4, 7, 8, 12, 17-19, 26, 30, 32, 34, 37, 38, and 42) were only detected in Meyer, and 5 spots (spots 15, 47-49 and 51) were only found in Diamond (Figure 5, Table 1). Meyer presented more protein spots that showed a change in abundance than Diamond,

**Table 1:** The 45 differentially accumulated proteins identified in the stolons of plants of the Meyer and Diamond genotypes subjected to cold stress for 28 d.

ID.	Accession no.	Protein name	PM	TMr/TpI	EMr/EpI	SC (%)	Genotype Meyer	Genotype Diamond
Redox homeostasis and defense response								
1	ABS70717, *Vigna* *angularis*	Pathogen-related protein	6	16.2/4.6	18.1/5.00	60	I	n
10	Q42443, *Oryza sativa*	Thioredoxin H-type	7	13.1/5.17	15.6/6.70	70	#0.46*±0.014§	n
13	CAAO6996, *Hordeum Vulgare*	Ascorbate peroxides (cytosolic)	12	26.8/5.54	20.4/6.31	50	0.45*±0.057	n
17	CAAO6996, *Hordeum Vulgare*	Ascorbate peroxidase (stromal)	10	27.5/5.85	26.8/5.34	27	3.82*±0.042	n
37	AAO37646, *Manihot esculenta*	NBS-LRR resistance protein RGH2	7	48.7/6.58	54.0/6.40	22	3.96*±0.179	n
42	AAP69615, *Oryza sativa*	Catalase	10	56.8/6.58	66.6/6.30	25	6.25*±0.059	n
Signal transduction								
6	ABG73446, *Oryza* *brachyantha*	RAB protein	6	24.7/5.71	15.5/6.00	45	0.42^*^±0.009	0.24^*^±0.042
34	NP193184,*Arabidopsis thaliana*	Protein kinase	16	50.9/5.80	52.1/6.02	37	2.66^*^±0.212	n
39	NP974162,*Arabidopsis thaliana*	F-box protein	11	57.7/5.65	59.6/5.70	28	0.43^*^±0.066	n
45	AAB05457, *Oryza sativa*	SNF1-related protein kinase	13	59.4/6.58	37.0/6.57	26	n	0.13^*^±0.005
47	NP001149914, *Zea mays*	Protein phosphatase 2c protein	9	44.9/5.04	46.4/5.84	28	n	17.2^*^±0.156
48	NP601152338, *Zea mays*	Auxin response factor 1	11	75.3/5.87	67.0/5.63	58	n	2.10^*^±0.009
Photosynthesis								
2	YP654221, *Oryza sativa*	Rubisco large subunit	8	29.7/6.45	18.2/5.10	27	I	n
7	YP762314, *Morus* *indica*	NADH-plastoquinone oxidoreductase subunit I	7	20.0/6.79	14.9/6.21	43	I	n
8	YP654221, *Oryza sativa*	Rubisco large subunit	10	29.7/6.45	15.9/6.50	33	I	n
16	BAD19812, *Triticum aestivum*	Rubisco small subunit	9	13.3/5.84	25.7/5.52	28	0.47^*^±0.005	n
32	AAT80326, *Arachnoides*	Rubisco large subunit	13	47.1/6.60	43.0/6.30	29	2.45^*^±0.123	n
52	BAB19812, *Triticum aestivum*	Rubisco small subunit	12	13.3/5.84	21.6/5.12	75	n	0
Energy metabolism								
3	NP194325,*Arabidopsis thaliana*	Vacuolar ATP synthase G3	7	12.1/5.11	16.0/5.12	67	0.30^*^±0.113	n
18	NP00105236, *Zea mays*	Cellulose synthase	6	22.0/6.25	26.5/5.50	39	I	n
19	AAM12449, *Oryza sativa*	ATPase α subunit	22	29.4/5.27	26.6/5.62	39	2.67^*^±0.113	n
28	NP195218, *Zea mays*	Pyruvate dehydronase	10	40.0/5.56	30.9/5.93	32	0.41^*^±0.075	n
29	ACJ71334, *Oryza* *nivara*	ADP-glucose pyrophosphorylase large subunit	12	58.2/5.55	40.6/6.24	24	2.87^*^±0.213	2.46^*^±0.067
30	NP178073, *Solanum tuberosum*	Phosphoglycerate kinase	10	52.0/6.07	38.6/6.80	23	14.13^*^±0.130	n
31	NP187884,*Arabidopsis thaliana*	Phosphoglycerate kinase	12	50.0/6.23	45.0/6.10	38	2.95^*^±0.174	3.82±0.013
33	AAC47193, *Oryza sativa*	Enolase	9	48.0/5.31	55.1/5.81	33	4.63^*^±0.052	I
38	AAC54522, *Campanula* *fragilis*	ATP synthase β subunit	10	58.4/6.8	58.4/6.81	31	3.21^*^±0.128	n
49	ACO48252, *Arachis hypogaea*	Phosphoenolpyruvate carboxylase	16	119.2/6.15	90.6/6.30	15	n	I
Other material metabolism								
11	AAP54710, *Oryza sativa*	Cytochrome P450 protein	11	29.9/5.90	18.1/6.59	46	0.40^*^±0.028	n
23	O48293, *Glycine max*	Cytochrome P450 protein	7	21.9/5.64	26.3/5.00	35	0.44^*^±0.047	n
4	AAP55038, *Arabidopsis thaliana*	Nucleoside diphosphate kinase	9	32.5/5.55	14.9/5.70	38	I	n
36	NP198367, *Arabidopsis thaliana*	Adenylate Kinase family protein	13	58.7/6.25	51.5/6.25	35	3.15^*^±0.038	2.47^*^±0.255
50	XP00287355, *Arabidopsis* *lyrata* *subsp.* *lyrata*	Dihydropyrimidinase	7	27.1/6.60	28.4/6.60	51	n	0.16^*^±0.004
20	CAD37971, *Arabidopsis thaliana*	Flavanone-3-hydroxylase	10	30.9/5.44	29.4/5.73	37	0.48^*^±0.002	n
25	CAA75996, *Zea mays*	Dihydroflavonol-4-reductase	10	39.9/5.88	36.4/5.10	20	0.49^*^±0.033	n
46	XP002868965, *Arabidopsis* *lyrata*	1-amino-cyclopropane-1-carboxylate synthase 8(ACS 8)	9	52.7/6.46	37.4/6.60	28	n	0.12^*^±0.024
14	AAB47602, *Oryza* *sativ*	Prolyl 4-hydroxylase, putative	10	32.0/5.81	26.0/6.30	51	0.17^*^±0.024	n
Protein biosynthesis								
26	NP001104874,*Arabidopsis thaliana*	Translation initiation factor	10	47.2/5.29	35.7/5.60	27	2.01^*^±0.180	n
40	O24310, *Pisum sativum*	Elongation factor Tu	7	53.1/6.62	58.8/6.42	22	3.62^*^±0.330	12.55^*^±0.024
Protein folding and assembly								
5	ACG24730, *Zea mays*	Prefoldin subunit 1	7	14.9/5.29	13.3/5.60	42	0.40^*^±0. 033	n
12	NP00149468, *Zea mays*	Proteasome α3 subunit	10	27.6/6.61	21.1/6.52	38	I	n
DNA replication								
22	ACS37303, *Murraya* Koenigii	PSF2	8	22.4/5.91	25.0/5.05	58	0.47^*^±0.024	n
Transcription								
43	BAB72061, *Oryza sativa*	bZIP transcription factor	10	36.5/6.47	24.5/6.50	38	0	n
Cellular process								
51	NP974162, *Arabidopsis thaliana*	Myosin heavy chain-related	14	68.6/5.36	67.0/5.70	27	n	3.66^*^±0.051
Unclassfied								
15	AAT76419, *Oryza sativa*	Expressed protein	8	26.9/5.86	21.7/5.52	32	0.41^*^±0.005	4.35^*^±0.599

ID, spot ID (corresponding to Figure 3); PM, the number of peptides matched; TpI and TMr, the theoretical isoelectric point and molecular mass, respectively; EpI and EMr, the experimental isoelectric point and molecular mass, respectively; SC, the sequence coverage of identified proteins; #, the protein intensity ratio (Treatment/Control); I, the spot is detected in the treatment but not in the control; n, the spot is not detected in 2-DE; 0, the spot is detected in the control but not in the cold treatment; * significant treatment effect (P<0.05) ; § standard deviation (STDEV) for fold change protein.

### Identification of differentially expressed proteins

**Figure 6:**
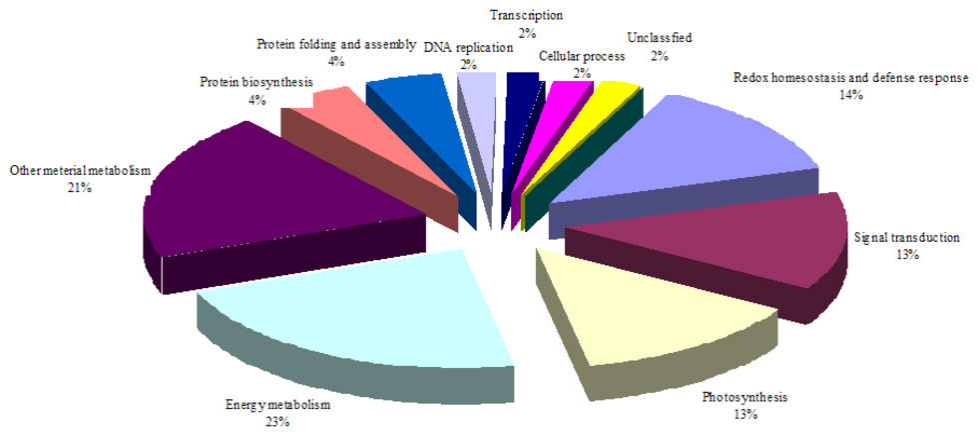
Functional grouping of the 45 identified proteins spots. Meyer and Diamond plants were treated with temperatures of 8/2 °C for 28 d, and the total zoysiagrass stolons proteins were then subjected to 2-DE analysis. The classification is based on KEGG pathway analysis (http://www.kegg.jp/kegg/pathway.html) and the literature.

Searches for 45 protein spots were performed using the Mascot search probability-based Mows score. The results of the database searches are shown in Table 1. Because little genomic and proteomic sequence information is available for *Zoysia* spp. Willd, the selected proteins were identified as being homologous to proteins of the other plant species present in the database (Table 1). The cold-responsive proteins were classified into 10 functional classes according to KEGG pathway analysis (http://www.kegg.Jp/kegg/pathway.html) and the literature (Figure 6 and Table 1); redox homeostasis (14%, spots 1, 10, 13, 17, 37 and 42); signal transduction (13%, spots 6, 34, 39, 45, 47 and 48); photosynthesis (13%, spots 2, 7, 8, 16, 32 and 52); energy metabolism (23%, spots 3, 18, 19, 28-31, 33, 38 and 49); other material metabolism (21%, spots 4, 11, 14, 20, 25, 23, 36, 46 and 50); protein biosynthesis (4%, spots 26 and 40); protein folding and proteolysis (4%, spots 5 and 12); DNA replication (2%, spot 22); transcription (2%, spots 43); and cellular processes (2%, spot 51). Based on the 45 identified protein spots, we proposed a cold stress-responsive protein network, composed of several different functional components that exhibits a balance between reactive oxygen species production and scavenging, accelerated protein biosynthesis and proteolysis, enhanced photosynthesis, abundant energy supply and enhanced biosynthesis of carbohydrates.

## Discussion

Many plants become more resistant to freezing temperatures when first exposed to low nonfreezing temperatures, a process known as cold acclimation or cold hardening [30]. The degree of freeze tolerance of *Arabidopsis thaliana* increased after a 4°C treatment [31]. In this study, the freeze tolerance under cold stress of Meyer and Diamond were analyzed using electrolyte leakage (EL) method, under cold treatment, the freeze tolerance of both Meyer and Diamond increased. To further study the molecule mechanism for the increasing of freeze tolerance in zoysiagrass stolons under cold stress, a proteomic approach was used. In this study, comparative analysis of the proteins accumulating in freezing-tolerant and freezing–sensitive species led to the identification of many differentially accumulated proteins. Some of these proteins have been well characterized regarding their response to cold and other stresses, but others have not been well studied with respect to their role in plant stress response.

In this study we employed silver staining to visualize proteins arrayed 2-DE. Silver staining was first presented as a tool for viualization of proteins in 2-DE gels in 1979 [32]. Silver staining is one of the most sensitive protein-staining methods and can detect proteins down to the nanogram level. However, it has the following disadvantages: difficulty in it application and a low repetition rate. Silver staining also has poor compatibility with mass-spectrometry (MS) compared to traditional Coomassie Blue staining as it includes glutaraldehyde in the sensitization solution [33]. Moreover, because it is not an endpoint procedure the process has to be stopped subjectively at the optimal time for quantification. This involves a decision by visual inspection, and thus is not very reliable for quantification [34]. In addition, the dynamic range is just two orders of magnitude, which is a limitation when working with complex protein mixtures [35]. Howerver, modern silver staining is no longer an erratic technique, the dispersion of the signals (a measure of variability) is not greater with silver staining than with Coomassie Blue. Many studies have replaced glutaraldehyde with formaldehyde in order to be compatible with MALDI-TOF analysis [36] or have used improved silver staining apparatus [37]. Grove et al. [38] providing a method to increase the dynamic range on a single 2-DE gel by using time resolved RGB images captured during gel color development. By calculating the max rate of silver stain change for each point on the gel and using this value instead of the absolute staining intensity the approach appears to allow for a larger dynamic range for 2-DE gel quantification. In order to improve the repeatability, several gels can be run for each sample and these stained at same time. In our study, all 12 gels were stained at the same time by using the modified apparatus developed by Sinha et al. [37]. This provided full compatibility with MALDI-TOF analysis and increased sensitivity and reproducibility.

### Identified proteins involved in antioxidant defense

Abiotic stresses, such as drought, cold and heat, can cause the accumulation of reactive oxygen species (ROS) to occur in plant cells. Plants develop ROS-scavenging strategies to control ROS levels and cope with oxidative stress through antioxidant defense systems, such as ascorbate peroxidase (APX), catalase (CAT), and thioredoxin H-type (Tr-h). Similar to what has been described in rice seedlings exposed to cold stress [1,8,39], a significant up-regulation of proteins involved in the response to oxidative stresses, such as APX (spot 17), CAT (spot 42) and Tr-h (spot 10), was expected only in Meyer plants. In this study, the abundance of APX (spot 13) was down-regulated 0.45 fold (P<0.05) compared with the control, whereas the abundance of APX (spot 17) was up-regulated 3.82 fold (P<0.05). The APX (spot 13) was identified as cytosolic APX, a different isoenzyme of APX from spot 17 (stromal APX), simultaneous up- and down-regulation in different APX isoenzymes were detected under cold stress (7, 8), which show that functionally different APXs respond under the cold and control conditions. However, further investigation is essential to confirm this hypothesis. This result indicates that Meyer exhibited a stronger ability to scavenge H_2_O_2_ than Diamond under cold stress.

Treatment with cold also significantly increased the abundance of a pathogen-related protein (PR, spot 1), NBS-LRR resistance protein RGH2 (spot 37) only in Meyer stolons (Table 1). To protect themselves from infection by microbial pathogens, plants have evolved a large number of immune receptors, such as PR, resistance protein and disease resistance proteins that sense pathogen-derived molecules and trigger a defense response [40]. Disease resistance proteins were found to be up-regulated in pea under chilling stress [41]. Wang et al. [12] observed an increase in the abundance of PR proteins in moss in response to cold-stress.

Taken together, our results suggest that cold stress enhances the accumulation of antioxidant enzyme and resistance-related proteins in the Meyer cultivar, which could be one reason that Meyer plants present better cold tolerance than Diamond plants.

### Identified protein involved in signal transduction

In this study, six protein spots, corresponding to a RAB protein (spot 6), protein kinase (PK, spot 34), f-box protein (spot 39), SNF1-related protein kinase (SnRK, spot 45), protein phosphatase 2c protein (PP2c, spot 47) and auxin response factor (ARF, spot 48) were identified in Meyer or Diamond stolons under cold stress. Previous studies have shown that all of these proteins, except ARF, play a critical role in plant responses to cold, drought, salt and light stress [8,12,42-45]. RAB (spot 6) was significantly (P<0.05) down-regulated in the stolons of both cultivars under cold stress whereas PK (spot 34) and f-box proteins (spot 39) were up-regulated in Meyer under cold stress, while cold stress decreased the abundance of SnRKs (spot 45) and increased the abundance of PP2c (spot 47) and ARF (spot 48) in Diamond stolons under cold stress. Yan et al. [8] observed a large increase in RAB levels in rice seedlings in response to chilling stress. Jain et al. [44] reported that the expression of an f-box protein-encoding gene was influenced by light, salt and cold stress, and the over-expression of an f-box protein gene reduced abiotic stress damage in rice [46]. SnRKs have been implicated in environmental stress [47], especially stresses related to drought and salt [43], through participating in ABA signaling [48]. Protein phosphatase 2Cs (PP2cs) belongs to the protein serine/threonine phosphatase family. PP2cs have been studied extensively and play roles in growth, development, and the response to hormones and abiotic stress [49], and Yan et al. [8] detected up-regulation of these phosphatases in rice seedlings under cold stress. Auxin response factors (ARFs) are transcriptional activators and repressors that bind with specificity to TGTCTC AuxREs in the promoters of primary/early auxin response genes [50]. The up-regulation of a protein kinase and down-regulation of an f-box protein observed in Meyer stolons under cold stress indicated that the calcium signaling pathway or MAPK signaling pathway and ethylene signaling pathway might be enhanced by cold stress in Meyer stolons. Additionally, the up-regulation of PP2C and ARF1 and down-regulation of an SNF1-related protein kinase detected in Diamond plants indicate that the auxin signaling pathway is stimulated, while the ABA signaling pathway is inhibited, in Diamond stolons under cold stress. The differences in signal transduction pathways observed between the Meyer and Diamond cultivars may explain why they present different cold tolerances.

### Identified proteins associated with photosynthesis

Plants may adjust their photosynthesis via gene regulation to adapt to cold environments, and the abundances of some proteins involved in the Calvin cycle were altered in the present study. Six proteins were identified in zoysiagrass stolons in this study, including the five Rubisco subunits, such as Rubisco large subunit (RLS, spots 2, 8 and 32), the Rubisco small subunit (RSS, spots 16 and 52), and NADH-plastoquinone oxidoreductase subunit 1 (Ndh, spot7). It has been demonstrated that Rubisco is highly regulated to control flux through the photosynthetic carbon reduction cycle in response to short-term fluctuations in the environment [51]. In our experiment, extended cold treatment up-regulated the abundance of one RLS proteins (spots 32) and induced new accumulation of two RLS (spot 2 and 8), in addition to down-regulating the abundance of one RSS protein (spot 16) in Meyer stolons. Gao et al. [52] found that 24 days of cold treatment decreased the abundance of RLS and caused heterogeneous changes in RSS in *Thellungiella* rosette leaves. RSS (spot 52) was only detected in Diamond stolons that were not subjected to cold treatment. The Ndh complex aids in the maintenance of non-photochemical quenching through setting the redox steady-state level of the intersystem carriers and then optimizing the rate of the cyclic electron flow in barley under ozone-mediated oxidative stress in the chloroplast thylakoid lumen [53]. In this study, Ndh (spot 7) was newly accumulated in Meyer stolons under cold stress. None of the RSS or Ndh proteins were identified in Diamond stolons under cold stress.

In summary, our results suggest that cold stress enhances photosynthesis in Meyer stolons through increasing Rubisco accumulation and PS II electron transport in the chloroplast. However, Rubisco accumulation was decreased in Diamond stolons under cold stress.

### Identified proteins implicated in the inhibition of folding and enhancement of biosynthesis and proteolysis

Gene expression can be regulated at the transcriptional, post-transcriptional, translational and post-translational levels [8]. In the present study, cold stress caused changes five proteins intensities in zoysiagrass stolons, and these proteins were divided into two groups: a protein biosynthesis group and a protein folding and proteolysis group (Table 1). The abundances of translation initiation factor (IF, spot 26) and elongation factor Tu (EF-Tu, spot 40), which function in protein biosynthesis, were up-regulated in zoysiagrass stolons under cold stress (Table 1). EF-Tu is a member of the GTP/GDP-binding proteins and interacts with various partners during the elongation cycle of protein biosynthesis [54]. Cui et al. [1] found that cold stress increases the EF-Tu accumulation in rice seedlings.

Two other proteins, prefoldin subunit 1 (spot 5) and the proteasome α3 subunit (spot 12), which are associated with protein folding and proteolysis, were identified in this study. Cold treatment decreased the abundance of prefoldin subunit 1 and induced the accumulation of the proteasome α3 subunit in Meyer stolons. Prefoldin is a cofactor of the group II chaperonins, this specific protein captures and stabilizes unfolded proteins and then delivers them to chaperonins for correct folding [55]. The proteasome degrades unneeded or damaged proteins through proteolysis [7] and functions in the stress response by removing abnormal proteins [56]. In the present experiments, cold treatment induced the accumulation of the proteasome α3 subunit in Meyer stolons. Thus, there may be an increased rate of protein turnover in Meyer in response to cold stress. This finding suggests that a high protein turnover rate occur because proteolysis is required for plants to eliminate misfolded polypeptides in a cold environment.

The present study has demonstrated that the inhibition of protein folding and enhancement of protein biosynthesis and proteolysis are required for zoysiagrass to survive to cold stress.

### Identified proteins associated with energy metabolism

In this study, 10 protein spots (3, 18, 19, 28, 29, 30, 31, 33, 38 and 49) associated with energy metabolism were observed in zoysiagrass stolons under cold stress. Seven of these protein spots are related to carbohydrate metabolism, the other two, the ATP synthase α subunit (ATPase α subunit, spot19) and the ATP synthase β subunit (ATPase β subunit, spot 38), are associated with ATP synthesis during oxidative phosphorylation in mitochondria the inner mitochondrial membrane, and vacuolar ATP synthase G3 (V-ATPase G3, spot 3), which is the proton pump to provide the energy for transport of ions and metabolites by acidifying compartments in the endomembrane.

Five proteins that are involved in the glycolysis pathway and TCA cycle, corresponding to pyruvate dehydrogenase (PDH, spot 21), phosphoglycerate kinase (PGK, spots 30 and 31), enolase (ENO, spot 33) and phosphoenolpyruvate carboxylase (PEPC, spot 49), were identified in this study. PGK, ENO, and PEPC have been previously associated with the cold stress response [1,7,57,58]. With the exception of PDH, the other four proteins were significantly up-regulated or induced in zoysiagrass stolons under cold treatment. Cold stress increased the abundance of PGK (spots 30 and 31) and ENO (spot 33) in Meyer stolons, whereas it induced the accumulation of ENO (spot 33) and PEPC (spot 49) and increased the abundance of PGK (spot 31) in Diamond stolons. Furthermore, the applied cold stress treatment decreased the abundance of PDH (spot 28) in Meyer stolons, and PDH E2 subunit breakdown has been observed in *Arabidopsis* under oxidative stress [59]. PEPC are crucial enzymes in the C_4_ cycle. PEPC catalyzes the combination of carbon dioxide with phosphoenolpyruvate to form oxaloacetate in the mesophyl. PEPC activities decline particularly dramatically during 10/7 °C treatments in *Z* *. japonica* [58]. In this study, PEPC was induced in Diamond stolons under cold stress. These difference may be associated the greater cold tolerance exhibited by Meyer plants compared Diamond plants.

Two of the identified proteins are involved in carbohydrate metabolism: cellulose synthase (spot 18) and the ADP-glucose pyrophosphorylase large subunit (ADPase, spot 29). Cellulose synthase plays an important role in the biosynthesis of cellulose in plants [60]. In this study, cold treatment induced the accumulation of cellulose synthase in Meyer stolons, which represents the first report of a response of cellulose synthase to cold stress. ADPase catalyzes the synthesis of ADP-glucose which is a glucosyl substrate that can be used for the synthesis of starch polymers. It is induced by 21 days of cold stress in *Festuca* *pratensis* [13] and up-regulated by Cu stress in rice [61]. In the present study, cold stress up-regulate the accumulation of ADPase in the stolons of Meyer and Diamond.

The ATPase α subunit (spot 19) and the ATPase β subunit (spot 38) participate in oxidative phosphorylation [62]. Previous studies have revealed changes in the abundance of ATPase in the leaves of rice seedlings [1], *Thellungiella* rosette leaves [52] and moss gametophores [12]. In our experiments, treatment with cold up-regulated the accumulation of the ATPase α subunit and ATPase β subunit in Meyer stolons, and a similar pattern of regulation was observed for these two proteins in rice seedlings exposed to cold stress [1,8].

In plants, V-ATPase has been localized to vacuoles and other membranes of the secretory system, including the endoplasmic reticulum (ER), Golgi and small vesicles as well as the plasma membrane. V-ATPase activity maintains the central vacuole pH at 5.5 and is required to provide energy for the transport of ions and organic metabolites [62]. Cold stress significantly decreased the accumulation of V-ATPase G3 (spot 3) in Meyer stolons, indicates that cold stress inhibited the V-ATPase activity and the concomitant decreases in proton motive force may affect solute compartmentation and possibly the hardiness of plants to low temperature.

The induction and increases of the identified proteins related to the glycolysis pathway, TCA cycle and oxidative phosphorylation observed in zoysiagrass stolons suggest that an abundant energy supply is required for zoysiagrass stolons to respond to cold stress. The energy supply in Meyer stolons is more abundant compared with Diamond stolons.

### Identified proteins involved in other types of metabolism and pathway

In this study, 13 proteins that are involved in primary metabolisms and other pathways, such as the metabolisms of lipids, nucleotides, amino acids, plant hormones or secondary metabolites, DNA replication, and cellular process, or that act as transcription factors, were detected.

Plant cytochromes P450s (CYPs) are ubiquitously distributed and form a superfamily of enzymes that are involved in the biosynthesis of plant hormones and secondary metabolites [63], and CYPs accumulate in response to cold stress [12]. In this study, the intensities of two protein spots (spots 11 and 23), which were identified as CYPs, were significantly decreased in Meyer stolons under cold stress. Our results suggested that lipid metabolism in zoysiagrass stolons is affected by cold stress.

Three proteins, a nucleotide diphosphate kinase (NDPK, spot 4), adenylate kinase family protein (ADK, spot 36) and dihydropyrimidinase (DH, spot 50), that are associated with nucleotide metabolism were also associated with the response to cold stress in zoysiagrass. NDPK and ADK have previously been identified as being involved in the response to cold stress [1,7,8,39,64]. In this study, cold stress induced the accumulation of NDPK in Meyer stolons and increased the ADK activity in the stolons of both cultivars, whereas the accumulation of DH in Diamond stolons decreased in response to stress. DH catalyzes the chemical reaction that converts 5,6-dihydrouracil to 3-ureidopropanoate in the second step in pyrimidine nucleotide metabolism to maintain pyrimidine homeostasis [65], this is the first report of DH regulation in response to cold stress. Our results indicate that nucleotide metabolism increases in zoysiagrass stolons under cold stress.

Protein spot 20 and 25 were identified respectively as flavanone-3-hydroxylase (F3H) and dihydroflavonol-4-reductase (DFR), respectively, which are related to the biosynthesis of secondary metabolites, and both proteins were markedly decreased in Meyer stolons under cold stress. F3H catalyzes an early step in flavonoid metabolism and is related to blight resistance [66] and drought stress [67]. DFR catalyzes the reduction of dihydroflavonols to leucoanthocyanidins in the anthocyanin pathway and it is a key enzyme [68]. This is the first report of F3H and DFR being associated with the response to cold stress. Decreases in secondary metabolite levels caused by F3H and DFR acting as a cold stress tolerance mechanism in plants requires further analysis.

Spot 14, which identified as prolyl 4-hydroxylase (PH), was decreased significantly in Meyer stolons by cold stress. PH catalyzes the formation of peptidyl 4-hydroxyproline from proline residues during arginine and proline metabolism [69].

One protein that was significantly down-regulated by cold stress in Diamond stolons in this study was 1-amino-cyclopropane-carboxylate synthase 8 (ACS8, spot 46). ACS is the rate-limiting enzyme in the ethylene biosynthetic pathway in plants. Cold induces the accumulation of ACS in apples [70], and Yamagami et al. [71] found that the ACS gene could also be induced by pathogen infection.

Cold treatment decreased the accumulation of PSF2 (spot 22, related to DNA replication) and the bZIP transcription factor (spots 43, related to transcription) in Meyer stolons, whereas it increased the accumulation of a myosin heavy chain-related protein (spot 51, related to cellular processes) in Diamond stolons. PSF2 is a subunit of the go-ichi-ni-san (GINS) complex, which localizes to DNA replication origins and has been implicated in the assembly of the DNA replication machinery [72]. The present study provides the first report of PSF2 accumulation being decreased by cold stress, indicating that DNA replication is inhibited in response to cold stress in the Meyer plants. The basic leucine zipper (bZIP) transcription factors are involved in various aspects of signaling in plants, such as light responses, hormone signaling [73] and pathogen defense [74]. Bae et al. [5] found that cold stress increased the expression bZIP transcription factors, whereas in the present study, bZIP transcription factors were not expressed in Diamond stolons under cold stress. Myosins comprise a family of ATP-dependent motor proteins that are best known for their role in muscle contraction and their involvement in a wide range of other eukaryotic motility process The abundance of myosin heavy chain-related proteins increased significantly in the Diamond plants under cold stress in the present study, and the increases in the intensity of myosin-like proteins have also been observed in rice seedlings [8]. Further investigation is necessary to understand the functions of myosin in the cold stress response.

### A possible cold stress-responsive protein network in zoysiagrass stolons

**Figure 7:**
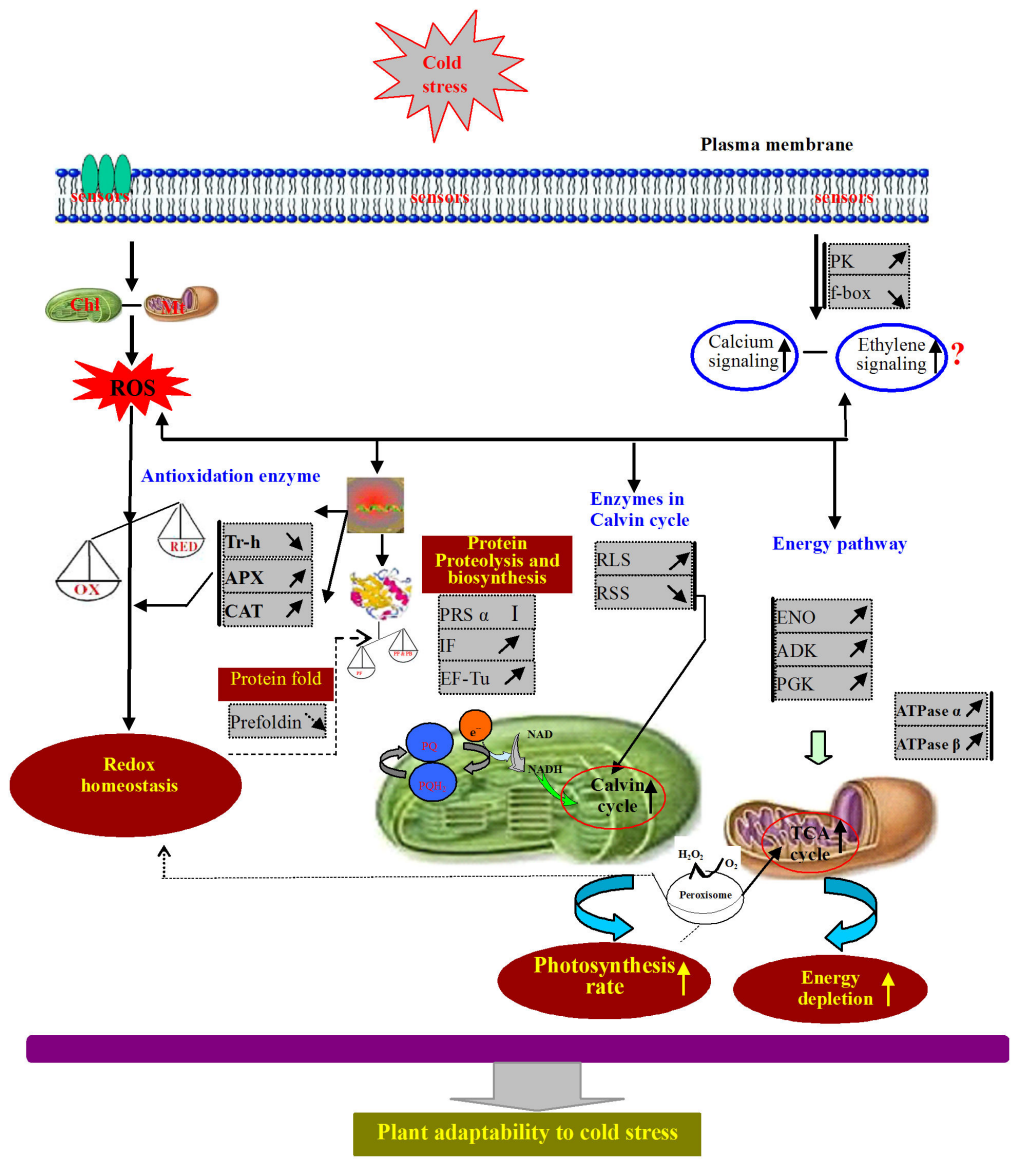
A-putative model of the cold-stress responses in zoysiagrass stolons. The cold-responsive proteins that were increased are indicated by “↗”, those that decreased by “↘”, and those proteins that were induced are indicated by “I”. See the text for details. Tr-h, thioredoxin H-type; APX, ascorbate peroxide; CAT, catalase; IF, translation initiation factor; EF-Tu, elongation factor Tu; PRS α, proteasome α_3_ subunit; RLS, Rubisco large subunit; RSS, Rubisco small subunit; Ndh, NADH–plastoquinone oxidoreductase subunit 1; ATPase α, ATP synthase α subunit; ATPase β, ATP synthase β subunit; ENO, enolase; ADK, adenylate kinase family protein; PGK, phosphoglycerate kinase; PEPC, phosphoenolpyruvate carboxylase; OX, oxidation; RED, reduction; PP, proteolytic protein; PB, protein biosynthesis; PF, protein fold.

In the present study, based on the obtained proteomics data, a cold stress-responsive protein network was proposed. This network comprises many of the 45 cold-responsive proteins that were identified in zoysiagrass stolons (Figure 7 and Table 1). This network consists of several functional components, including components that are involved in signaling pathway, balancing ROS production and scavenging, accelerated protein biosynthesis and reduced protein folding, enhanced photosynthesis, the provision of abundant energy supplies and enhanced biosynthesis of carboxydrates and nucleotides. The development of this network allows us to better understand and describe possible management strategies related to the cellular activities that occur in cold stress-treated zoysiagrass stolons.

Under cold stress, zoysiagrass stolons can perceive cold stress signals through putative sensors and transmit them to the cellular machinery by enhancing Ca^2+^ signal transduction pathway by regulation of RAB, enhancing the MAPK signal transduction pathway by re-regulation of PGK or enhancing the ethylene signal transduction by down-regulation of F-box. On the other hand, from a homeostasis-based view, mitochondria and chloroplasts are involved to re-establish a new homeostasis in energy, primary metabolites and redox balance. First, ROS production is increased by long time cold stress, which could lead to redox imbalance in the cells of zoysiagrass stolons. To counteract the damage caused by ROS increases in cells, zoysiagrass stolons are expected to attempt to achieve a redox balance through enhancing the accumulation of antioxidant enzymes (e.g., APX and CAT) to reduce the cellular ROS content to a normal level. Second, under cold stress, photosynthesis in zoysiagrass stolons is impaired through induction or up-regulation of RLS, down-regulation of RSS and induction of Ndh involved in Calvin cycle. Third, protein biosynthesis and proteolysis are enhanced in zoysiagrass stolons in response to cold stress through up-regulation of IF and EF-Tu and induction of while protein folding is inhibited through the down-regulation of prefoldin subunit 1. Fourth, the energy supply of zoysiagrass stolons were enhanced to response in cold stress by up-regulation NADH-plastoquinone oxidoreductase subunit 1, the ATPase α subunit, the ATPase β subunit, ENO and PGK, induction of cellulose synthesis, which are involved in the glycolysis pathway and TCA cycle. Fifth, except above changes to response to cold stress, the plant hormones or secondary metabolites and DNA replication of the zoysiagrass stolons were inhibited by down-regulation of CYPs, F3H, DFR and PSF, nucleotide metabolism is enhanced though induction of NDPK and up-regulation of ADK. Through these main changes in signal transduction, photosynthesis, metabolic pathways as well as redox balancing, zoysiagrass stolons strive to adapt to and/or resist external cold stress.

Furthermore, the cold-tolerant Meyer cultivar exhibits a greater ROS scavenging ability, more abundant energy supplies, and increased photosynthesis, protein synthesis and proteolysis compared with the cold-sensitive Diamond cultivar under cold stress, which may partly explain why Meyer is more cold tolerant than Diamond.

## Conclusions

In conclusion, the abundances of many protein spots related to photosynthesis, energy metabolism, protein biosynthesis and proteolysis were increased or induced under long-term cold stress, to a greater extent in freeze-tolerant Meyer than in freeze-sensitive Diamond. Antioxidant defense proteins associated with ROS scavenging, such as APX, CAT and Tr-h, were increased under cold stress in Meyer, suggesting that these proteins may contribute to cold tolerance in this zoysiagrass. Our results suggest that the superior freeze tolerance observed in *Zoysia*. spp. Willd. plants could mainly be associated with the maintenance of proteins that are involved in photosynthesis (Rubisco large subunit), protein biosynthesis and proteolysis (IF, EF-Tu and proteasome α3 subunit), energy metabolism (NADH-plastoquinone oxidoreductase subunit 1, ATPase α subunit, ATPase β subunit, ENO and ADKs) and antioxidant defense (APX, CAT and Tr-h). Further analyses will be conducted to confirm the accumulation patterns of these cold-responsive proteins via western blotting and to investigate the enzyme activities of certain proteins that exhibit differential expression between cold-tolerant and cold-sensitive genotypes. Such information will provide further insights into the biological functions of these proteins related to cold tolerance.

## Supporting Information

Figure S1 **Raw 2-DE gels of Meyer and Diamond.** The two varieties were subjected to cold treatment (and control). A total of three replicates were conducted.
(TIF)
