# Supplementary figures and images for: Comparative Proteomic Analysis of the Stolon Cold Stress Response between the C4 Perennial Grass Species Zoysia japonica and Zoysia metrella
Source: PLoS One. 2013 Sep 26;8(9):e75705. doi: 10.1371/journal.pone.0075705 (PMC3784457; doi:10.1371/journal.pone.0075705)

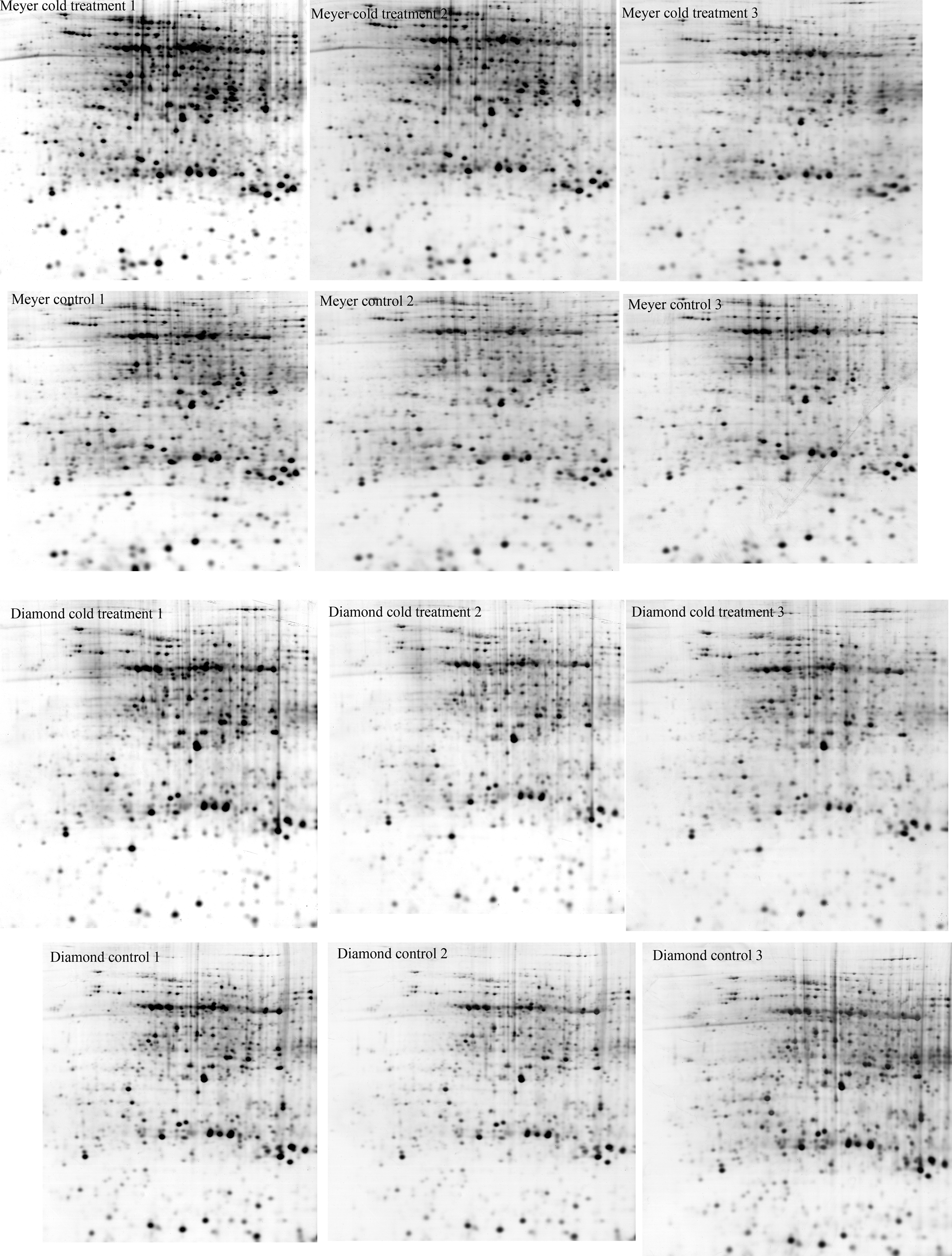

Supplement: Figure S1 — Raw 2-DE gels of Meyer and Diamond. The two varieties were subjected to cold treatment (and control). A total of three replicates were conducted. (TIF) [file pone.0075705.s001.tif]
